# Sex Differences in the Association of Serum Albumin and Cholesterol with Cognitive Function in Older Adults with Cognitive Impairment

**DOI:** 10.1002/alz70861_108239

**Published:** 2025-12-23

**Authors:** DERONG ZENG, Maolu Yin, Xiaoyu Hong, Teruaki Kawasaki, Ichiro Akiguchi, Ayae Kinoshita

**Affiliations:** ^1^ kyoto university, kyoto, kyoto Japan; ^2^ Dana Farber Boston Children’s Cancer and Blood Disorders Center, Harvard Medical School, Boston, MA USA; ^3^ Division of Endocrinology, Peking University Shenzhen Hospital, Shenzhen, Guangdong China; ^4^ Kyoto Clinical and Translational Research Center for Neurocognitive Disorders, Kyoto Japan; ^5^ Kyoto Clinical and Translational Research Center for Neurocognitive Disorders, kyoto, kyoto Japan; ^6^ Kyoto University Graduate School of Medicine, Kyoto Japan

## Abstract

**Background:**

The contribution of nutritional and metabolic factors to cognitive decline remains underexplored. Emerging evidence suggests that biomarkers such as serum albumin, cholesterol, and HbA1c may influence cognitive health in aging populations. This study examined the associations between key blood biomarkers and cognitive function in older adults with cognitive impairment, with attention to potential sex‐specific effects.

**Methods:**

This cross‐sectional study included 438 older adults (mean age = 81.9 years; 47.5% male) who visited a dementia care center in Japan between 2018 and 2022. Cognitive function was assessed using the Mini‐Mental State Examination (MMSE). Blood biomarkers analyzed included total protein, albumin, creatine kinase, cholesterol, triglycerides, and HbA1c. Multivariable linear and logistic regression models were conducted, adjusting for age, sex, and education. Interaction effects and sex‐stratified models were used to identify gender differences.

**Results:**

Higher education (β = 0.395, *p* < 0.001) and albumin levels (β = 1.74, *p* = 0.045) were positively associated with MMSE scores, while cholesterol was inversely associated with cognition (β = −0.013, *p* = 0.045). Logistic regression showed that albumin was associated with reduced odds of cognitive impairment (OR = 0.53, 95% CI: 0.29–0.97). Sex‐stratified analyses revealed that the inverse association between cholesterol and MMSE was significant in females (p = 0.022) but not in males. HbA1c was not significantly associated with cognitive function overall, though a non‐significant trend toward a positive association was observed in men. No significant interaction between age and HbA1c was found.

**Conclusions:**

Serum albumin and cholesterol are independently associated with cognitive function among older adults with cognitive impairment. Notably, the effect of cholesterol appears to differ by sex, with stronger cognitive impacts observed in women. These findings emphasize the clinical relevance of metabolic and nutritional biomarkers in dementia risk assessment and suggest a potential for gender‐specific strategies in cognitive health interventions.

